# Factors Associated With Coronary Artery Disease Progression Assessed By Serial Coronary Computed Tomography Angiography

**DOI:** 10.5935/abc.20170049

**Published:** 2017-05

**Authors:** Gabriel Cordeiro Camargo, Tamara Rothstein, Maria Eduarda Derenne, Leticia Sabioni, João A. C. Lima, Ronaldo de Souza Leão Lima, Ilan Gottlieb

**Affiliations:** 1 Casa de Saúde São José; Rio de Janeiro, RJ, Brazil; 2 Centro de Diagnóstico por Imagem CDPI, Rio de Janeiro, RJ, Brazil; 3 Johns Hopkins University, Baltimore, USA; 4 Hospital Universitário Clementino Fraga Filho - Universidade Federal do Rio de Janeiro (UFRJ); Rio de Janeiro, RJ - Brazil; 5 Instituto Nacional de Cardiologia, Rio de Janeiro, RJ - Brazil

**Keywords:** Coronary Artery Disease/physiopathology, Coronary Amgiography, Tomography, X-Ray Computed, Percutaneous Coronary Intervention

## Abstract

**Background:**

Coronary computed tomography angiography (CCTA) allows for noninvasive coronary artery disease (CAD) phenotyping. Factors related to CAD progression are epidemiologically valuable.

**Objective:**

To identify factors associated with CAD progression in patients undergoing sequential CCTA testing.

**Methods:**

We retrospectively analyzed 384 consecutive patients who had at least two CCTA studies between December 2005 and March 2013. Due to limitations in the quantification of CAD progression, we excluded patients who had undergone surgical revascularization previously or percutaneous coronary intervention (PCI) between studies. CAD progression was defined as any increase in the adapted segment stenosis score (calculated using the number of diseased segments and stenosis severity) in all coronary segments without stent (in-stent restenosis was excluded from the analysis). Stepwise logistic regression was used to assess variables associated with CAD progression.

**Results:**

From a final population of 234 patients, a total of 117 (50%) had CAD progression. In a model accounting for major CAD risk factors and other baseline characteristics, only age (odds ratio [OR] 1.04, 95% confidence interval [95%CI] 1.01-1.07), interstudy interval (OR 1.03, 95%CI 1.01-1.04), and past PCI (OR 3.66, 95%CI 1.77-7.55) showed an independent relationship with CAD progression.

**Conclusions:**

A history of PCI with stent placement was independently associated with a 3.7-fold increase in the odds of CAD progression, excluding in-stent restenosis. Age and interstudy interval were also independent predictors of progression.

## Introduction

Coronary artery disease (CAD) is the worldwide leading cause of death.^1^ Clinical and revascularization approaches have been shown to decrease the morbidity and mortality from chronic CAD. Despite treatment, the clinical course of chronic CAD usually consists of progression of atherosclerosis punctuated by flares of unpredictable clinical events.^2,3^ In a meta-analysis, Cannon et al. have shown that patients with previous documented CAD on secondary prophylaxis with high-dose statins in addition to contemporary clinical management still have a 7% incidence of composite events and 2% mortality per year.^4^ Although CAD is a progressive inflammatory and degenerative disorder,^5,6^ some studies have demonstrated the feasibility of interruption or even regression of atherosclerosis progression, as measured by invasive techniques such as intravascular ultrasound^7,8^ and optical coherence tomography.^9^ Previous studies have identified markers of anatomical atherosclerosis progression, but these studies were restricted to patients submitted to percutaneous coronary intervention (PCI) undergoing repeat invasive coronary angiography (ICA), as part of the study protocol.^10-12^

Coronary computed tomography angiography (CCTA) is able of noninvasively phenotyping CAD in a broader range of clinical scenarios and provides good diagnostic performance for obstructive CAD detection, as well as strong prognostic information.^13-15^ A recent meta-analysis has shown a high correlation between CCTA and measures of plaque burden and stenosis severity derived by intracoronary ultrasound.^16^ Able of depicting disease even with minimal luminal narrowing, CCTA offers an opportunity to track incipient CAD and obstructive coronary stenosis.

In the present study, we sought to identify the variables associated with CAD progression on sequential CCTA testing in patients with and without previous PCI.

## Methods

### Subjects

Of 5055 clinically indicated CCTAs performed in 4607 patients in our institution between December 2005 and March 2013, we identified 382 individuals who underwent sequential testing at least 90 days apart. A total of 72 patients who had undergone surgical revascularization were excluded, since CAD progression in these cases may have been associated with diversion of the flow from the bypasses and not necessarily with the usual pathophysiology of atherosclerosis.^17^ Additionally, 76 patients who had undergone PCI between CCTA studies were also excluded, since the quantification of the progression of native vessel disease would be biased by the artificial improvement of the treated segment. The remaining 234 patients comprised the study sample. Before each test, information on medication use, CAD risk factors, and previous coronary events and stress testing results were obtained during an interview with a physician. Baseline characteristics were established for each subject at the time of the first CCTA exam.

Each patient gave a written informed consent for inclusion of their information into our database, including clinical data and test results that were personally recorded by the physician responsible for the pretest interview and by another one in charge of the study reporting, respectively. For this study, as in every other involving this data source, access to the database by research personnel could only be made by a query, which returns a renumbered spreadsheet filled with the requested data, excluding identifying information such as patient's name and record number. Since no personal information was disclosed, institutional review board approval was not requested for this study. None of the authors of this paper was responsible for treating the patients included in this analysis or in the database in general.

### CCTA imaging technique

CCTA studies were performed on a 256-slice scanner (Brilliance iCT, Philips Healthcare, Cleveland, Ohio) or one of two 64-slice computed tomography scanners (Brilliance 64, Philips Healthcare, Cleveland, Ohio, USA and Somatom Sensation 64, Siemens Healthcare, Erlangen, Germany) during contrast injection, using a bolus tracking technique aiming at acquiring images at peak coronary opacification. Prospective electrocardiogram (ECG) triggering was strongly encouraged in examinations performed on scanners with this feature. When unavailable or not recommended (*i.e.*, irregular heart rate [HR]), retrospective ECG gating was used instead.

All patients with a baseline HR above 60 bpm were given oral (100 mg) and/or intravenous (5-20 mg) metoprolol to achieve a prescanning HR of 60 bpm or less. Sublingual isosorbide dinitrate 0.4 mg was administered 3-5 minutes prior to the contrast image acquisition, unless contraindicated.

### CCTA analysis

**Figure 1:**
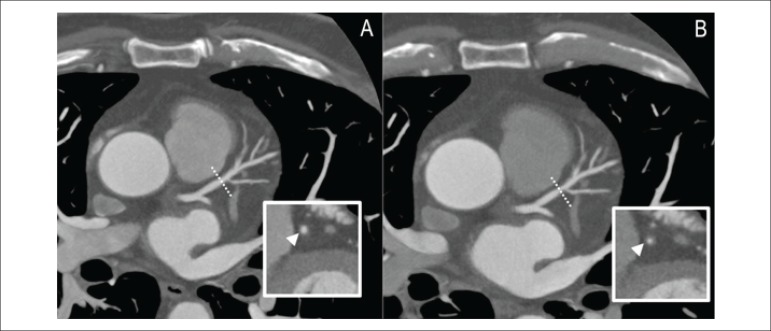
Coronary artery disease (CAD) progression on coronary computed tomography angiography (CCTA) in a 58-year-old male presenting a very mild CAD in the proximal left anterior descending coronary artery at baseline (A). Evident disease progression is seen at 13 months at the same site, with moderate luminal stenosis (B) best appreciated in the vessel's transverse plane (arrowhead).

All exams were blindly reviewed by a single cardiac imaging expert (I.G.). The coronary artery tree was divided into 15 segments,^18^ and coronary atherosclerosis was defined as at least 1 mm^2^ of tissue structure that could be individualized within or adjacent to the lumen and differentiated from pericardial and epicardial tissue, as previously described.^18^ The extent and severity of the CAD were assessed using an adapted version of the segment stenosis score (SSS), which has been previously described and validated as a strong prognostic marker.^15^ Briefly, each of the 15 coronary segments was assigned a score from 0 to 4 based on the presence of atherosclerosis and degree of luminal narrowing: 0 (no atherosclerosis), 1 (1-29%), 2 (30-49%), 3 (50-69%), and 4 (70-100%). Scored segments were then added together to provide a final score ranging from 0 to 60. A progressing lesion, as seen on CCTA, is shown in Figure 1.

### CAD progression definition and treatment of stented segments

The SSS from the first and second CCTA studies were calculated, and disease progression was defined as any increase in SSS from baseline to follow-up CCTA. Conversely, regression was defined as any decrease in SSS from baseline to follow-up. Stented segments were excluded from disease progression or regression calculations. For multivariable adjustments of CAD severity at baseline, each stented segment was graded as a 70-100% stenosis aiming to overestimate baseline CAD severity in patients with stents. This baseline overestimation in stented patients was done in order to increase their disease severity and, since baseline SSS was included in the multivariable model, minimize the impact of the stent acting as a marker of more "aggressive" CAD presentation.

### Statistical analysis

Continuous variables are presented as mean ± standard deviation (SD) or median (interquartile range [IQR]), as appropriate. Categorical variables are presented as frequencies and percentages. Intergroup comparisons were analyzed using unpaired Student's *t* test or Mann-Whitney U test for continuous variables, as appropriate, and chi-square test for categorical variables. Univariable and stepwise backward multivariable logistic regression were used to assess individual predictors of CAD progression. A secondary multivariable analysis was performed in patients with evidence of atherosclerosis at baseline to identify independent predictors of CAD regression. Statistical significance was defined as a two-tailed p value below 0.05. All analyses were performed using SPSS 19.0 (SPSS Inc., Chicago, Illinois, USA).

## Results

**Figure 2:**
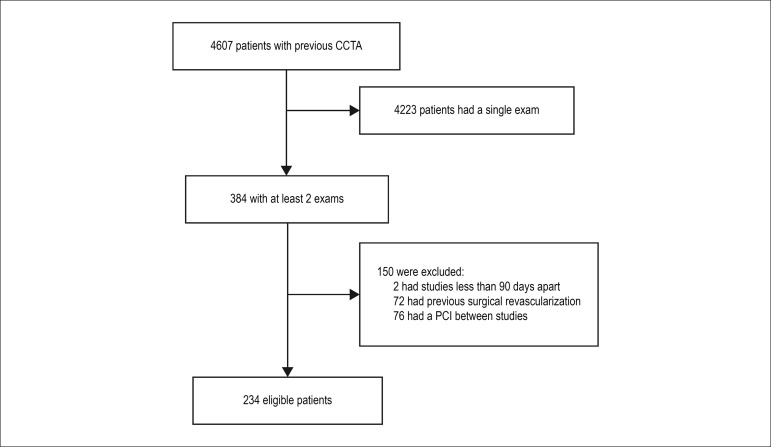
Flowchart of patient selection. The final study population comprised individuals with sequential coronary computed tomography angiography (CCTA) testing conducted at least 90 days apart and free of percutaneous coronary intervention (PCI) between studies or previous surgical coronary revascularization.

The study included 234 patients with a mean age of 60 ± 11 years, 79% of whom were males. The flowchart in Figure 2 shows the selection of the population. A total of 8% of the patients had a history of myocardial infarction, and 11% of them had a recent (less than 30 days before the index study) positive stress test result. A previous PCI had been conducted in 50 (21%) subjects, who had a total of 83 stented segments (mean of 1.7 per subject). Other baseline characteristics are summarized in Table 1.

**Table 1:** Patients’ baseline characteristics

Patients, n	234
Age (years), mean ± SD	59.8 ± 10.7
Male sex, n (%)	186 (79)
BMI (kg/m2), mean ± SD	27.7 ± 3.9
Exam interval (months), median (IQR)	32.4 (19.2 - 49.7)
Baseline SSS, median (IQR)	6 (2 - 11)
**Clinical risk factors**	
Hypertension, n (%)	117 (50)
Diabetes, n (%)	30 (13)
Dyslipidemia, n (%)	125 (53)
Family history CAD, n (%)	99 (42)
Glucose intolerance, n (%)	10 (4)
Current smoker, n (%)	25 (11)
Past smoker, n (%)	55 (24)
Positive stress test, n (%)	26 (11)
Previous MI, n (%)	18 (8)
Previous PCI, n (%)	50 (21)
**Medication use**	
Beta-blockers, n (%)	35 (15)
ACEI/ARB, n (%)	45 (19)
Antiplatelet, n (%)	46 (20)
Statin, n (%)	59 (25)

SD: standard deviation; BMI: body mass index; SSS: segment stenosis score; IQR: interquartile range; CAD: coronary artery disease; MI: myocardial infarction; PCI: percutaneous coronary intervention; ACEI: angiotensin-converting enzyme inhibitor; ARB: angiotensin receptor blocker.

During CCTA acquisition, the subjects' mean HR was 54 ± 7 bpm. The median radiation exposure was 4.7 mSv (4-6.4 mSv), and prospective ECG triggering was used in 79% of all studies. Of all exams, 35 (0.01%) segments were deemed unevaluable and were excluded from the analysis in both studies.

At baseline CCTA, 41 (17%) patients had no evidence of coronary atherosclerosis, while the CAD severity was deemed very mild (1-29%) in 60 (26%), mild (30-49%) in 65 (28%), moderate (50-69%) in 37 (16%), and severe (≥ 70%) in 31 (13%). The baseline SSS was 0 in 41 (17%) subjects, between 1 and 5 in 76 (32%) subjects, between 6 and 10 in 55 (24%) subjects, between 11 and 15 in 25 (11%) subjects, and 16 or above in 37 (16%) subjects.

The follow-up study was conducted at a median of 32 months (19-50 months) when 117 (50%) patients presented CAD progression.

Univariable logistic regression including all baseline characteristics revealed that age, interstudy interval, baseline SSS, and previous PCI were predictors of CAD progression. Table 2 lists the patients' characteristics according to CAD progression status. After multivariable adjustment, age, interstudy interval, and previous PCI emerged as independent predictors of progression. An independent 3.7-fold increased odds of progression was associated with a history of coronary stenting, as shown in Table 3.

**Table 2:** Patients’ baseline characteristics according to progression status

	All subjects		
	No Progression	Progression	p value
Patients, n	117	117	
Age (years), mean ± SD	58.3 ± 10.7	61.3 ± 10.8	0.03
Male sex, n (%)	90 (77)	96 (82)	0.42
BMI (kg/m^2^), mean ± SD	27.2 ± 3.9	28.0 ± 4.0	0.11
Exam interval (months), median (IQR)	29.8 (18.8 - 42.8)	34.1 (20.4 - 55.2)	0.05
Baseline SSS, median (IQR)	5 (1 - 9)	8 (2 - 14)	0.01
**Clinical risk factors**			
Hypertension, n (%)	58 (50)	59 (50)	1.00
Diabetes, n (%)	15 (13)	15 (13)	1.00
Dyslipidemia, n (%)	65 (56)	60 (51)	0.60
Family history CAD, n (%)	54 (46)	45 (38)	0.29
Glucose intolerance, n (%)	3 (3)	7 (6)	0.33
Current smoker, n (%)	16 (14)	9 (8)	0.20
Past smoker, n (%)	23 (20)	32 (27)	0.22
Positive stress test, n (%)	14 (12)	12 (10)	0.84
History of MI, n (%)	6 (5)	12 (10)	0.22
Previous PCI, n (%)	15 (13)	35 (30)	0.002
**Medication use**			
Beta-blockers, n (%)	18 (15)	17 (15)	1.00
ACEI/ARB, n (%)	21 (18)	24 (21)	0.74
Antiplatelet, n (%)	18 (15)	28 (24)	0.14
Statin, n (%)	31 (26)	28 (24)	0.76

SD: standard deviation; BMI: body mass index; SSS: segment stenosis score; IQR: interquartile range; CAD: coronary artery disease; MI: myocardial infarction; PCI: percutaneous coronary intervention; ACEI: angiotensin-converting enzyme inhibitor; ARB: angiotensin receptor blocker.

**Table 3:** Predictors of coronary artery disease (CAD) progression

	Univariable analysis			Multivariable analysis		
	Odds Ratio	95%CI	p value	Odds Ratio	95%CI	p value
Age (years)	1.03	1.00 - 1.05	0.03	1.04	1.01 – 1.07	0.01
Male sex	1.37	0.72 - 2.60	0.33	1.92	0.92 – 3.98	0.08
BMI (kg/m^2^)	1.06	0.99 - 1.13	0.12	1.07	0.99 – 1.15	0.08
Study interval (months)	1.01	1.00 - 1.03	0.02	1.03	1.01 – 1.04	< 0.001
Baseline SSS	1.04	1.01 – 1.09	0.02			
**Clinical risk factors**						
Hypertension	0.97	0.58 – 1.61	0.90			
Diabetes	1.00	0.46 – 2.15	1.00			
Dyslipidemia	1.19	0.71 – 1.99	0.51			
Family history CAD	1.37	0.82 – 2.31	0.23			
Glucose intolerance	0.41	0.10 – 1.64	0.21			
Current smoker	1.90	0.80 – 4.49	0.14			
Former smoker	0.65	0.35 – 1.20	0.17			
Positive stress test	1.19	0.53 – 2.69	0.68			
Previous MI	0.47	0.17 – 1.31	0.15			
Previous PCI	2.90	1.48 - 5.68	< 0.001	3.66	1.77 – 7.55	< 0.001
**Medication use**						
Beta-blockers	1.07	0.52 – 2.19	0.85			
ACEI/ARB	0.85	0.44 – 1.63	0.62			
Antiplatelet	0.58	0.30 – 1.12	0.10			
Statin	1.15	0.63 – 2.07	0.65			

95%CI: 95% confidence interval; SD: standard deviation; BMI: body mass index; SSS: segment stenosis score; IQR: interquartile range; CAD: coronary artery disease; MI: myocardial infarction; PCI: percutaneous coronary intervention; ACEI: angiotensin-converting enzyme inhibitor; ARB: angiotensin receptor blocker.

**Figure 3:**
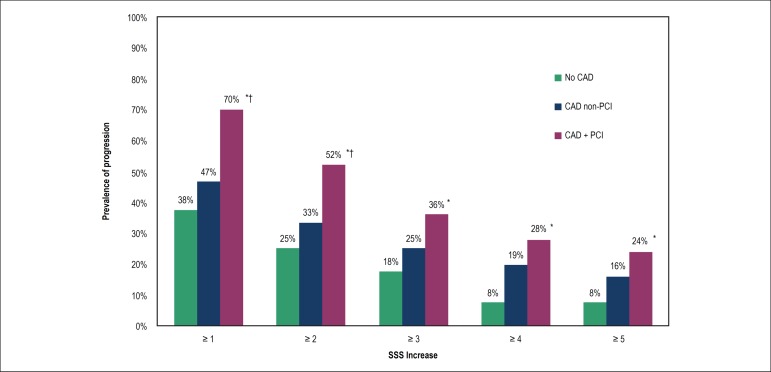
Prevalence and severity of segment stenosis score (SSS) increase according to subgroup. *p < 0.05 between coronary artery disease (CAD) + percutaneous coronary intervention (PCI) and no CAD; †p < 0.05 between CAD + PCI and CAD non-PCI.

Overall, 70% of the patients with previous PCI presented CAD progression, compared with 47% of those with baseline CAD but no stents (p = 0.003) and 38% without any CAD at baseline (p = 0.002). This higher rate of progression among PCI patients remained across a wide range of SSS increases, as shown in Figure 3. Differences in baseline characteristics among patients with and without stents are shown in Table 4.

**Table 4:** Patients’ baseline characteristics according to history of percutaneous coronary intervention (PCI)

Patients, n	non-PCI	PCI	p value
	184	50	
Age (years), mean ± SD	58.9 ± 11.1	63.4 ± 9.1	0.01
Male sex, n (%)	149 (81)	42 (74)	0.35
BMI (kg/m^2^), mean ± SD	27.7 ± 3.8	27.4 ± 4.4	0.56
Exam interval (months), median (IQR)	33.4 (22.0 - 53.1)	26.9 (15.0 - 37.2)	< 0.01
Baseline SSS, median (IQR)	4 (1 - 8)	16 (10 - 21)	< 0.001
**Clinical risk factors**			
Hypertension, n (%)	91 (49)	26 (52)	0.87
Diabetes, n (%)	20 (11)	10 (20)	0.10
Dyslipidemia, n (%)	89 (48)	36 (72)	< 0.01
Family history of CAD, n (%)	79 (43)	20 (40)	0.75
Glucose intolerance, n (%)	6 (3)	4 (8)	0.23
Current smoker, n (%)	22 (12)	3 (6)	0.31
Past smoker, n (%)	39 (21)	16 (32)	0.13
Positive stress test, n (%)	18 (10)	8 (16)	0.21
History of MI, n (%)	2 (1)	16 (32)	< 0.001
CAD progression, n (%)	82 (45)	35 (70)	< 0.001
**Medication use**			
Beta-blockers, n (%)	21 (11)	14 (28)	0.01
ACEI/ARB, n (%)	31 (17)	14 (28)	0.10
Antiplatelet, n (%)	18 (10)	28 (56)	<0.001
Statin, n (%)	38 (21)	21 (42)	<0.01

SD: standard deviation; BMI: body mass index; SSS: segment stenosis score; IQR: interquartile range; CAD: coronary artery disease; MI: myocardial infarction; PCI: percutaneous coronary intervention; ACEI: angiotensin-converting enzyme inhibitor; ARB: angiotensin receptor blocker.

On secondary analysis considering only subjects with evidence of CAD at the baseline CCTA (n = 193), disease regression was independently related only with a history of PCI with stent (OR 0.28, 95% confidence interval [95%CI] 0.10-0.77, p = 0.01), baseline SSS (OR 1.10, 95%CI 1.04-1.16, p = 0.01), and interstudy interval (OR 0.98, 95%CI 0.96-0.99, p = 0.02) on multivariable logistic regression.

## Discussion

In spite of medical and invasive treatments, CAD remains a progressive disease. Several studies reveal a high incidence of events among patients submitted to guideline-based optimal therapies, underlying the limitations of currently available therapeutic approaches.^19-21^ Angiographic CAD progression may identify subjects at a higher risk for cardiovascular events since plaque growth entails inflammatory activity and increased risk of rupture.^22^ The identification of predictors of CAD progression is epidemiologically important and allows a better understanding of the pathophysiology of CAD.

Our cohort consisted of "real world" patients, with and without previous evidence of CAD, including those with a history of PCI. Subjects with intervening PCI procedures between CCTA studies were excluded in order to avoid the bias of decreased stenosis due to stent placement. Similarly, previously implanted stented coronary segments were excluded from the progression analysis so that restenosis would not contaminate the results. In this setting, we found a 50% rate of native vessel (non-stented) CAD progression over a median follow-up of 32 months, which is in the upper range of previous studies using ICA.^23-29^ This may have been a result of the use of CCTA, which is capable of depicting three-dimensionally the coronary wall and is, therefore, not constrained by two-dimensional projections.

In multivariable analysis, age, interval between studies, and previous PCI were independent predictors of CAD progression. Specifically, previous PCI with stent placement, a potentially modifiable patient characteristic, was associated with a 3.7-fold increased odds of disease progression. Although this is the first study to our knowledge to show it using this technology, absolute causality between stent placement and progression cannot be made due to the retrospective and observational nature of this study. One potential bias could be that stents are only but a marker of faster progressing atherosclerosis biology. We vigorously tried to minimize this bias by adjusting the results to baseline CAD and other major risk factors, previous myocardial infarction and by overestimating the CAD burden for stented segments at baseline CCTA. Interestingly, a history of PCI was not only independently related to increased odds of disease progression, but also a 72% reduction in the odds of regression.

Most previous research on coronary atherosclerosis progression has focused on patients undergoing ICA in preparation for PCI, but they also are subject to bias.^10,20,21^ Without comparing CAD progression between PCI and non-PCI patients, potential effects of the invasive treatment on disease evolution cannot be derived. Nevertheless, even in this setting, two previous studies of subjects undergoing PCI have reported that a history of PCI before study entry was a significant and independent predictor of worse outcomes.^10,30^

Borges et al.^26^ reported results from a study comparing subjects undergoing medical treatment alone *versus* PCI in regards to native vessel CAD progression using ICA.^26^ The authors found that patients with a previous PCI had an independent 2.1-fold increased odds of CAD progression over 5 years when compared with those without prior PCI.

### Limitations

Since this was a retrospective and observational study, we are unable to establish with certainty a causality between PCI and CAD progression, although we judiciously tried to adjust the model for potential confounders. Despite the biases and given the paucity of research on this subject, this study generates questions that should be answered with large prospective randomized studies.

To determine the occurrence of CAD progression, we used the results of CCTA, which has lower spatial and temporal resolution than ICA.^31^ This fact may result in artifacts that hinder the CAD quantification. Although some inaccuracies may occur with this method, mostly related to stenosis overestimation, all patients were equally subjected to the same errors. Despite this limitation, the use of CCTA may offer some advantages in eccentric coronary plaque visualization and mild luminal narrowing.

Due to the limited number of subjects in our study, some questions remain to be answered by future investigations, such as the impact of gender and race on CAD progression, the relevance of the number of stented segments, differences in progression between bare metal and drug-eluting stents and, the most important of all, if this observed progression may translate into future events.

## Conclusion

In a "real world" population of patients referred to sequential CCTA testing, age and history of coronary artery stenting were independent predictors of native CAD progression, while the degree of baseline CAD assessed by SSS was not independently associated with this endpoint.

## References

[1] World Health Organization (2011). Global status report on noncommunicable diseases 2010.

[2] Alderman EL, Kip KE, Whitlow PL, Bashore T, Fortin D, Bourassa MG (2004). Native coronary disease progression exceeds failed
revascularization as cause of angina after five years in the Bypass
Angioplasty Revascularization Investigation (BARI). J Am Coll Cardiol.

[3] Alexopoulos D, Xanthopoulou I, Davlouros P, Damelou A, Mazarakis A, Chiladakis J (2010). Mechanisms of nonfatal acute myocardial infarction late after
stent implantation: the relative impact of disease progression, stent
restenosis, and stent thrombosis. Am Heart J.

[4] Cannon CP, Steinberg B A, Murphy S A, Mega JL, Braunwald E (2006). Meta-analysis of cardiovascular outcomes trials comparing
intensive versus moderate statin therapy. J Am Coll Cardiol.

[5] Epstein F, Ross R (1999). Atherosclerosis : an inflammatory disease. N Engl J Med.

[6] Stary HC, Chandler AB, Glagov S, Guyton JR, Insull W, Rosenfeld ME (1994). A definition of initial, fatty streak, and intermediate lesions
of atherosclerosis. A report from the Committee on Vascular Lesions of the
Council on Arteriosclerosis, American Heart Association. Circulation.

[7] Nissen S, Tuzcu E (2004). Effect of intensive compared with moderate lipid-lowering therapy
on progression of coronary atherosclerosis. JAMA.

[8] Nissen SE, Nicholls SJ, Sipahi I, Libby P, Raichlen JS, Ballantyne CM (2006). Effect of very high-intensity statin therapy on regression of
coronary atherosclerosis: the ASTEROID trial. JAMA.

[9] Diletti R, Garcia-Garcia HM, Gomez-Lara J, Brugaletta S, Wykrzykowska JJ, van Ditzhuijzen N (2011). Assessment of coronary atherosclerosis progression and regression
at bifurcations using combined IVUS and OCT. JACC Cardiovasc Imaging.

[10] Glaser R, Selzer F, Faxon DP, Laskey WK, Cohen H A, Slater J (2005). Clinical progression of incidental, asymptomatic lesions
discovered during culprit vessel coronary intervention. Circulation.

[11] Cutlip DE, Chhabra AG, Baim DS, Chauhan MS, Marulkar S, Massaro J (2004). Beyond restenosis: five-year clinical outcomes from
second-generation coronary stent trials. Circulation.

[12] Leon MB, Allocco DJ, Dawkins KD, Baim DS (2009). Late clinical events after drug-eluting stents: the interplay
between stent-related and natural history-driven events. JACC Cardiovasc Interv.

[13] Miller JM, Rochitte CE, Dewey M, Arbab-Zadeh A, Niinuma H, Gottlieb I (2008). Diagnostic performance of coronary angiography by 64-row
CT. N Engl J Med.

[14] Bamberg F, Sommer WH, Hoffmann V, Achenbach S, Nikolaou K, Conen D (2011). Meta-analysis and systematic review of the long-term predictive
value of assessment of coronary atherosclerosis by contrast-enhanced
coronary computed tomography angiography. J Am Coll Cardiol.

[15] Min JK, Shaw LJ, Devereux RB, Okin PM, Weinsaft JW, Russo DJ (2007). Prognostic value of multidetector coronary computed tomographic
angiography for prediction of all-cause mortality. J Am Coll Cardiol.

[16] Fischer C, Hulten E, Belur P, Smith R, Voros S, Villines TC (2013). Coronary CT angiography versus intravascular ultrasound for
estimation of coronary stenosis and atherosclerotic plaque burden: a
meta-analysis. J Cardiovasc Comput Tomogr.

[17] Dimitrova KR, Hoffman DM, Geller CM, Dincheva G, Ko W, Tranbaugh RF (2012). Arterial grafts protect the native coronary vessels from
atherosclerotic disease progression. Ann Thorac Surg.

[18] Min JK, Dunning A, Lin FY, Achenbach S, Al-Mallah MH, Berman DS (2011). Rationale and design of the CONFIRM (COronary CT Angiography
EvaluatioN For Clinical Outcomes: An InteRnational Multicenter)
Registry. J Cardiovasc Comput Tomogr.

[19] Mancini GBJ, Hartigan PM, Bates ER, Sedlis SP, Maron DJ, Spertus J A (2011). Angiographic disease progression and residual risk of
cardiovascular events while on optimal medical therapy: observations from
the COURAGE Trial. Circ Cardiovasc Interv.

[20] Zellweger MJ, Kaiser C, Jeger R, Brunner-La Rocca H-P, Buser P, Bader F (2012). Coronary artery disease progression late after successful stent
implantation. J Am Coll Cardiol.

[21] Yin Z-X, Zhou Y-J, Liu X-L, Han H-Y, Yang S-W (2011). Clinical predictors for progression of nonintervened nonculprit
coronary lesions despite low-density lipoprotein cholesterol less than 1.8
mmol/l after successful stent implantation. Coron Artery Dis.

[22] Sanz J, Fayad ZA (2008). Imaging of atherosclerotic cardiovascular disease. Nature.

[23] Rozenman Y (1997). Long-term angiographic follow-up of coronary balloon angioplasty
in patients with diabetes mellitusa clue to the explanation of the results
of the BARI study. J Am Coll Cardiol.

[24] Stone PH, Saito S, Takahashi S, Makita Y, Nakamura S, Kawasaki T (2012). Prediction of progression of coronary artery disease and clinical
outcomes using vascular profiling of endothelial shear stress and arterial
plaque characteristics: the PREDICTION Study. Circulation.

[25] Rozenman Y, Gilon D, Welber S (1995). Influence of coronary angioplasty on the progression of coronary
atherosclerosis. Am J Cardiol.

[26] Borges JC, Lopes N, Soares PR, Góis AFT, Stolf NA, Oliveira SA (2010). Five-year follow-up of angiographic disease progression after
medicine, angioplasty, or surgery. J Cardiothorac Surg.

[27] Lichtlen PR, Nikutta P, Jost S, Deckers J, Wiese B, Rafflenbeul W (1992). Anatomical progression of coronary artery disease in humans as
seen by prospective, repeated, quantitated coronary angiography. Relation to
clinical events and risk factors. The INTACT Study Group. Circulation.

[28] Bruschke a V, Kramer JR, Bal ET, Haque IU, Detrano RC, Goormastic M (1989). The dynamics of progression of coronary atherosclerosis studied
in 168 medically treated patients who underwent coronary arteriography three
times. Am Heart J.

[29] Alderman E, Corley S, Fisher L, Chaitman B, Faxon D, Foster E (1993). Five-year angiographic follow-up of factors associated with
progression of coronary artery disease in the coronary artery surgery study
(CASS). J Am Coll Cardiol.

[30] Stone GW, Maehara A, Lansky AJ, de Bruyne B, Cristea E, Mintz GS (2011). A prospective natural-history study of coronary
atherosclerosis. N Engl J Med.

[31] Arbab-Zadeh A, Hoe J (2011). Quantification of coronary arterial stenoses by multidetector CT
angiography in comparison with conventional angiography methods, caveats,
and implications. JACC Cardiovasc Imaging.

